# Supplementary material for: Anti-inflammatory therapy with nebulized dornase alfa for severe COVID-19 pneumonia: a randomized unblinded trial
Source: eLife. 2024 Jul 16;12:RP87030. doi: 10.7554/eLife.87030 (PMC11251720; doi:10.7554/eLife.87030)
Supplement: Supplementary file 1. — (A) Dexamethasone administration prior to recruitment Duration of dexamethasone treatment prior to recruitment and initiation of dornase alfa treatment in randomized and contemporary control participants. (B) Secondary endpoints in randomized participants only: Time to discharge, D-dimer, lymphocyte counts and procalcitonin measurements. (C) Secondary clinical endpoints in in randomized and contemporary control participants: Admission to ICU rates, length of stay in ICU, time on oxygen over 7- and 35 days follow-up, duration of mechanical ventilation, and proportion of individuals with superadded bacterial pneumonia. (D) Safety Table depicting the reported adverse events, the degree of severity and the relationship to dornase alfa therapy. (E) Cumulative Summary Tabulations of Serious Adverse Events. Serious adverse effects in randomized R-BAC and R-BAC +DA participants separated by system order class (infections, respiratory, thoracic and mediastinal disorders and vascular disorders). A total of 6 events were reported, with 2 in the R-BAC and 4 in the R-BAC +DA groups. [file elife-87030-supp1.docx]

**Supplementary File 1 - Supplementary Tables**

Supplementary File 1A: Duration of dexamethasone treatment prior to recruitment and initiation of dornase alfa treatment

|  | **R-BAC+DA (N=30)** | **R-BAC (N=9)** | **CC-BAC**  **(N=60)** | **T-BAC (N=69)** | **Total (N=99)** |
| --- | --- | --- | --- | --- | --- |
| **Length of Dexamethasone at baseline (days)** |  |  |  |  |  |
| N | 30 | 8 | 60 | 68 | 98 |
| Mean | 0.67 | 1 | 1.38 | 1.34 | 1.13 |
| SD | 0.76 | 1.20 | 0.64 | 0.73 | 0.79 |
| Median | 1 | 0.5 | 1 | 1 | 1 |
| Min | 0 | 0 | 0 | 0 | 0 |
| Max | 3 | 3 | 3 | 3 | 3 |

Supplementary File 1B. Secondary endpoints in randomised participants only

|  | **R-BAC+DA** | **R-BAC** | **Difference** | **p-value*** |
| --- | --- | --- | --- | --- |
| **Time to discharge (days)** |  |  |  |  |
| Number discharged | 27 | 8 | 19 |  |
| Median time to discharge** (95% CI) | 6  (4 to 7) | 4  (2 to-n.a.) | 2 |  |
| Hazard ratio*** (95% CI) |  |  | 1.18 (0.53 to 2.69) | 0.62 |
| **D-dimer (ug/L) FEU** |  |  |  |  |
| N | 28 | 6 |  |  |
| Least-squares mean (log)*  (95% CI) | 6.37  (6.01 to 6.74) | 7.55 (  6.71 to 8.39) | -1.18  (-2.02 to -0.33) | 0.008 |
| Least-square mean**  (95% CI) | 586.87 (407.44 to 845.31) | 1903.82  (821.57 to 4411.69) | 0.31  (0.13 to 0.72) |  |
| **Lymphocyte count (×10^9^/L)** |  |  |  |  |
| N | 30 | 9 |  |  |
| Least-squares mean (log)*  (95% CI) | -0.06  (-0.25 to 0.12) | -0.46  (-0.82 to -0.1) | 0.4  (0.03 to 0.76) | 0.033 |
| Least-square mean**  (95% CI) | 0.94  (0.78 to 1.13) | 0.63  (0.44 to 0.9) | 1.49  (1.03 to 2.13) |  |
| **Procalcitonin count (ng/ml)** |  |  |  |  |
| N | 26 | 7 |  |  |
| Least-square mean*  (95% CI) | 0.18  (-0.2 to 0.56) | 1.31  (0.56 to 2.05) | -1.13  (-1.88 to -0.37) | 0.005 |

*From log-rank test with treatment as a stratification variable.

**Estimated from Kaplan-Meier curve.

***From Cox proportional hazard model, adjusting for age, baseline CRP and treatment.

Supplementary File 1C. Secondary clinical endpoints

| **Admission to ICU over 7 days of follow up** |
| --- |

| N | R-BAC+DA n=30 | T-BAC n=69 |  |  |  |
| --- | --- | --- | --- | --- | --- |
|  | 23.3% | 21.74% |  |  | p=0.866 |
| **Length of ICU stay** | | | | | |
| LSM | 21.25 h | 19.85h |  |  | p=0.883 |
| 95% CI | 4.65-37.84 | 8-31.7 |  |  |  |
| **Admission to ICU over 35 d follow up** | | | | | |
| N | 23% | 23.19 |  |  | p=0.983 |
| LSM | 55.21 h | 60.6 h |  |  | P=0.905 |
| 95% CI | -23.59-134.00 h | 4.34-116.86 h |  |  |  |
|  |  |  |  |  |  |

| **Time on Oxygen over 7 days follow-up (hours)** | | | | | | | | | | |  |  |
| --- | --- | --- | --- | --- | --- | --- | --- | --- | --- | --- | --- | --- |
| N | 30 | | 69 | | |  | | |  | |  |  |
| Least-square mean* (95% CI) | 94.32 (72.86 to 115.79) | | 88.96 (73.64 to 104.29) | | | 5.36 (-18.92 to 29.65) | | | 0.662 | |  |  |
| **Time on Oxygen over 35 days follow-up (hours)** | | | | | | | | | | |  |  |
| N | 30 | | 69 | | |  | | |  | |  |  |
| Least-square mean* (95% CI) | 133.22 (52.01 to 214.43) | | 156.35 (98.36 to 214.33) | | | -23.12 (-115.02 to 67.77) | | | 0.618 | |  |  |
| **Proportion of individuals on mechanical ventilation over 7 days follow-up** | | | | | | | | | | | |  |
| N (%) | 5 (16.67) | 9 (13.04) | | | -4 (3.62) | | |  | | | |  |
| Odds ratio* (95%CI) |  |  | | | 1.36 (0.39 to 4.66) | | | 0.628 | | | |  |
| **Proportion of individuals on mechanical ventilation over 35 days follow-up** | | | | | | | | | | | |  |
| N (%) | 5 (16.67) | 9 (13.04) | | | -4 (3.62) | | |  | | | |  |
| Odds ratio* (95% CI) |  |  | | | 1.36 (0.39 to 4.66) | | | 0.628 | | | |  |
| *From Logistic regression model, adjusted for age, sex, BMI, baseline CRP, serious condition and treatment. | | | | | | | | | | | |  |
| **Proportion of individuals with Superadded Bacterial Pneumonia over 7 days follow-up** | | | | | | | | | | | |  |
| N (%) | 1 (3.33) | | | 3 (4.35) | | |  | | |  | | |
| Odds ratio* (95% CI) | 0.9 (0.08 to 10.21) | | |  | | |  | | | 0.934 | | |
| **Proportion of individuals with Superadded Bacterial Pneumonia over 35 days follow-up** | | | | | | | | | | | | |
| N (%) | 2 (6.67) | | | 3 (4.35) | | |  | | |  | | |
| Odds ratio* (95% CI) | 1.81 (0.26 to 12.61) | | |  | | |  | | | 0.548 | | |
| *From Logistic regression model, adjusted for age, sex, BMI, baseline CRP, serious condition, and treatment. | | | | | | | | | | | | |

Supplementary File 1D. Safety

| **Subject** | **R-BAC+DA or R-BAC only** | **Adverse event** | **Serious?** | **Relationship to study drug** |
| --- | --- | --- | --- | --- |
| COV002 | Dornase-alfa + BAC | Cough & SOB | No | Not related |
| COV003 | Dornase-alfa + BAC | Mild depression | No | Not related |
| COV003 | Dornase-alfa + BAC | Mild cognitive impairment | No | Not related |
| COV005 | Dornase-alfa + BAC | Struggle to sleep | No | Not related |
| COV005 | Dornase-alfa + BAC | Transaminitis (ALT 91 - NR 10-35 iu/L) | No | Not related |
| COV005 | Dornase-alfa + BAC | Constipation | No | Not related |
| COV007 | Dornase-alfa + BAC | Blood stain in sputum | No | Not related |
| COV012 | Dornase-alfa + BAC | Small Pericardial Effusion | No | Not related |
| COV012 | Dornase-alfa + BAC | Dysphonia | No | Not related |
| COV012 | Dornase-alfa + BAC | Hypercapnia | No | Not related |
| COV013 | Dornase-alfa + BAC | Ulcerative Colitis flare | No | Not related |
| COV013 | Dornase-alfa + BAC | Bradycardia | No | Not related |
| COV015 | Dornase-alfa + BAC | Mechanical Fall | No | Not related |
| COV015 | Dornase-alfa + BAC | Dizziness | No | Not related |
| COV018 | Dornase-alfa + BAC | Dehydration | No | Not related |
| COV018 | Dornase-alfa + BAC | Lower Respiratory Tract Infection | No | Not related |
| COV018 | Dornase-alfa + BAC | Haemoptysis | No | Not related |
| COV020 | Dornase-alfa + BAC | Chest pain | No | Not related |
| COV022 | Dornase-alfa + BAC | Microcytic anaemia | No | Not related |
| COV022 | Dornase-alfa + BAC | Elevated Blood glucose | No | Not related |
| COV023 | Dornase-alfa + BAC | Tachypnoea (PR 32BPM) | No | Not related |
| COV023 | Dornase-alfa + BAC | Hyperglycaemia (BM 14.9) | No | Not related |
| COV031 | Dornase-alfa + BAC | Chest Pain | No | Not related |
| COV035 | Dornase-alfa + BAC | Left leg spasm | No | Not related |
| COV035 | Dornase-alfa + BAC | Rectal bleed due to haemorrhoids | No | Not related |
| COV037 | Dornase-alfa + BAC | Chest Pain | No | Not related |
| COV002 | Dornase-alfa + BAC | Tingling of the mouth | No | Definitely |
| COV035 | Dornase-alfa + BAC | Headache | No | Unlikely |

Supplementary File 1E: Cumulative Summary Tabulations of Serious Adverse Events (SAEs)

| **System Organ Class (SOC)**  Preferred Term | **Total** | |
| --- | --- | --- |
|  | **Dornase alfa (IMP) arm** | **Best Available Care (control) arm** |
| **Infections and infestations**  Pyelonephritis | 1  1 |  |
| **Respiratory, thoracic and mediastinal disorders**  Aspiration pneumonia  Hospital acquired pneumonia  Organising pneumonia  Pulmonary embolism | 3  1  1  1 | 1  1 |
| **Vascular disorders**  Acute subdural haematoma |  | 1  1 |
| **Total** | 4 | 2 |
