# Supplementary material for: Anti-inflammatory therapy with nebulized dornase alfa for severe COVID-19 pneumonia: a randomized unblinded trial
Source: eLife. 2024 Jul 16;12:RP87030. doi: 10.7554/eLife.87030 (PMC11251720; doi:10.7554/eLife.87030)
Supplement: Supplementary file 3. [file elife-87030-supp3.docx]

**Customer:** University College London (UCL)

**Information Type:** Statistical Analysis Plan v1.6

| Title: A single-site, randomised, controlled, parallel design, open-label investigation of an approved nebulised recombinant human DNase enzyme (dornase alfa) to reduce hyperinflammation in hospitalised participants with COVID-19 (The COVASE trial)  Effective Date: | Statistical Analysis Plan v1.6 for 132333  12-AUG-2021 |
| --- | --- |

**Description:** Statistical analysis plan for UCL’s COVID-19 trial, COVASE.

## Author’s Name, Title and Functional Area:

Jamie Inshaw, Senior Statistician, Strategic consulting

## Approved by:


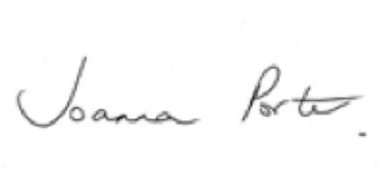


Prof. Joanna Porter Date: 16^th^ August 2021 Chief Investigator

UCL


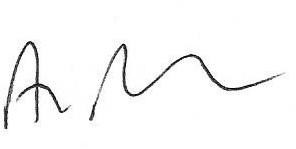


Aiden Flynn Date: 12^th^ August 2021 Director

Exploristics Limited

Copyright 2021 Exploristics Limited. All rights reserved. Unauthorised copying or use of this information is prohibited.

| **Audit Trail For This Document** | | | |
| --- | --- | --- | --- |
| **Compiled by/Affiliation** | **Type of Change** | **Date** | **Version** |
| Jamie Inshaw, Exploristics | Creation | 29OCT2020 | 1.0 |
| Jamie Inshaw, Exploristics | Edits following discussions with the client. | 06NOV2020 | 1.1 |
| Jamie Inshaw, Exploristics | Accepted client re- wording and changed the maximum permitted number of individuals to  48 (not 52). | 16DEC2020 | 1.2 |
| Jamie Inshaw, Exploristics | Changed from propensity score matching at the interm to inclusion of all eligible historical controls.  Included mock interim tables.  Changed “number of comorbidities” in the adjustment and matching analysis to “any key  comorbities”. | 04JAN2021 | 1.3 |
| Jamie Inshaw, Exploristics | Updated to expand the details of the primary and secondary analyses in preparation for the final  analysis | 29JAN2021 | 1.4 |
| Jamie Inshaw, Exploristics | Updated the mechanical ventilation and ventilator- associated pneumonia analysis, as incidence cannot be reliably inferred from the data. Clarified how “time on oxygen” will be calculated. Clarified which covariates will be removed from models if convergence fails or over-  fitting present. | 14JUL2021 | 1.5 |
| Jamie Inshaw, Exploristics | Removed oxygen use summary by day as data available in COVASE and HC participants not sufficient for the analysis to be performed.  Removed HCs from the | 12AUG2021 | 1.6 |

|  | WHO ordinal scale  analysis, as not available in HCs. |  |  |
| --- | --- | --- | --- |
| Effective Date |  | 12AUG2021 | 1.6 |

**Table of Contents**

[ABBREVIATIONS 6](#_bookmark0)

1. [INTRODUCTION 7](#_bookmark1)
2. [STUDY OBJECTIVE(S) AND ENDPOINT(S) 8](#_bookmark2)
   1. [Study Objective(s) 8](#_bookmark3)
   2. [Study Endpoint(s) 8](#_bookmark4)
      1. [Primary Endpoint 8](#_bookmark5)
      2. [Secondary Endpoints 8](#_bookmark6)
      3. [Exploratory Endpoints 9](#_bookmark7)
   3. [Statistical Hypotheses 9](#_bookmark8)
      1. [Primary null hypothesis 9](#_bookmark9)
      2. [Secondary null hypotheses 9](#_bookmark10)
      3. [Exploratory null hypotheses 9](#_bookmark11)
3. [STUDY DESIGN 10](#_bookmark12)
4. [PLANNED ANALYSES 12](#_bookmark13)
5. [SAMPLE SIZE CONSIDERATIONS 12](#_bookmark14)
6. [ANALYSIS POPULATIONS 13](#_bookmark15)
   1. [Primary analysis population (Intention to treat) 13](#_bookmark16)
   2. [Per protocol population 13](#_bookmark17)
   3. [Comparator population 13](#_bookmark18)
   4. [Safety population 13](#_bookmark19)
   5. [Exploratory analysis population 13](#_bookmark20)
7. [TREATMENT COMPARISONS 14](#_bookmark21)
8. [GENERAL CONSIDERATIONS FOR DATA ANALYSES 14](#_bookmark22)
   1. [Dependent Variables (Endpoint Variables) 14](#_bookmark23)
   2. [Independent Variables 14](#_bookmark24)
   3. [Examination of Subgroups 14](#_bookmark25)
      1. [Primary endpoint analyses 14](#_bookmark26)
      2. [Secondary endpoint analyses 15](#_bookmark27)
   4. [Multiple Comparisons and Multiplicity 15](#_bookmark28)
9. [DATA HANDLING CONVENTIONS 15](#_bookmark29)
   1. [Premature Withdrawal and Missing Data 15](#_bookmark30)
   2. [Derived and Transformed Data 15](#_bookmark31)
10. [DESCRIPTIVE STATISTICS 15](#_bookmark32)
    1. [Disposition of Subjects 15](#_bookmark33)
    2. [Demographic and Baseline Characteristics 15](#_bookmark34)
11. [ANALYSIS CORRESPONDING TO THE STUDY OBJECTIVES 16](#_bookmark35)
    1. [Primary endpoint analysis 16](#_bookmark36)
    2. [Secondary endpoint analyses 18](#_bookmark37)
    3. [Exploratory endpoint analyses 20](#_bookmark38)
    4. [Safety analyses 20](#_bookmark39)
12. [INTERIM ANALYSIS 20](#_bookmark40)
13. [REFERENCES 21](#_bookmark41)
14. [ATTACHMENTS 22](#_bookmark42)
    1. [Table of Contents for Data Display Specifications 22](#_bookmark43)

# ABBREVIATIONS

| AE | Adverse Event |
| --- | --- |
| APACHE | Acute physiology score + age points + chronic health points |
| BAC | Best Available Care |
| BID | Twice per day |
| CF | Cystic Fibrosis |
| cfDNA | Cell-free DNA |
| CI | Chief Investigator |
| CoV | Coronavirus |
| CRF | Case Report Form |
| CRP | C-reactive protein |
| DNA | Deoxyribonucleic acid |
| DNase | Deoxyribonuclease |
| EudraCT | European Clinical Trials Database |
| FDA | Federal drug administration |
| FVC | Forced vital capacity |
| H3 | Histone 3 |
| ICU | Intensive care unit |
| IL-1 β | Interleukin -1 beta |
| IL-6 | Interleukin-6 |
| IL-8 | Interleukin-8 |
| ISARIC | International Severe Acute Respiratory and Emerging Infection  Consortium |
| MPO | Myeloperoxidase |
| MV | Mechanical Ventilation |
| NIHR | National Institute for health research |
| NETs | Neutrophil extracellular traps |
| PCR | Polymerase chain reaction |
| PCT | ProCalcitonin |
| PD | Pharmacodynamics |
| QD | Once per day |
| RECOVERY | Randomised evaluation of COVID-19 therapy |
| RCT | Randomised Controlled Trial |
| SAP | Statistical Analysis Plan |
| SAE | Serious Adverse Event |
| SOFA | Sepsis-related Organ Failure Assessment |
| SOP | Standard Operating Procedure |
| TNFα | Tumour necrosis factor alpha |
| TEAE | Treatment Emergent Adverse Event |
| UCL | University College London |
| UCLH | University College London hospital |
| VAP | Ventilator-Associated Pneumonia |
| WHO | World health organisation |

# INTRODUCTION

The clinical spectrum of SARS-CoV-2 infection (COVID-19) appears to be wide, encompassing asymptomatic infection, mild upper respiratory tract illness (majority of cases) and severe viral pneumonia with respiratory failure and even death in the minority of subjects. In severe viral pneumonia excessive and inappropriate activation of neutrophils can result in the formation of neutrophil extracellular traps (NETs) which exacerbate the clinical course of the pneumonia. These NETs consist of DNA, histones and other components of neutrophils (e.g. myeloperoxidase (MPO)). These NETs are found in the lungs and in the circulation and contribute to organ damage. A treatment that reduces NET formation is likely to reduce the exuberant inflammatory response and thereby improve the clinical course of viral pneumonia and save lives.

NETs have been shown to drive disease in influenza pneumonia as well as in subjects with cystic fibrosis (CF). In addition, their role has been explored in various pre-clinical models of viral infection (mouse and bovine) and shown that reduction of the NETs improves symptoms and increases survival. High neutrophil infiltration is prominent in the lungs of COVID-19 patients and evidence of NET components in the circulation and lung biopsies has been reported in clinical study of COVID-19 patients.

Dornase alfa is a recombinant human DNase I that has been approved since 1994 for the treatment of CF. It is delivered directly to the lungs by nebulisation and has been shown to:

- reduce NETs and inflammation
- reduce the relative risk of developing a respiratory tract infection
- improve pulmonary function in both chronic and acute exacerbation of inflammatory CF

Dornase alfa is safe and well-tolerated in children and adults with CF at doses ranging from 2.5mg QD up to a maximum of 10mg BID.

This study proposes to treat hospitalised subjects with COVID-19 by administration of 2.5mg dornase alfa BID for 7 days. The effect on NETs, inflammation and clinical course will be closely monitored.

We expect to see a reduction in circulating NETS and inflammatory biomarkers that will result in clinical benefit. Historic controls will be obtained from an existing database of 120 subjects with COVID-19 that have been admitted to UCL since the beginning of the outbreak.

It is worth noting that dornase alfa can be self-administered at home. Therefore, dornase alfa has the potential to provide benefit in subjects with COVID-19 who have mild disease and are self- isolating and in those discharged from hospital to recuperate at home.

This statistical analysis plan (SAP) describes the key elements of the data handling and analysis strategy that generates evidence of the efficacy and safety of dornase alfa in participants with COVID-19 from the data collected in the study.

# STUDY OBJECTIVE(S) AND ENDPOINT(S)

## Study Objective(s)

**Primary objective**: to assess the effect of dornase alfa on C-reactive Protein (CRP) in hospitalised participants with COVID-19.

**Secondary objective**: to assess the effect of dornase alfa on clinical responses in hospitalised participants with COVID-19.

**Exploratory objective**: to assess the effect of dornase alfa on inflammation, biomarkers of NETs, coagulation, complement activation and haemolysis in hospitalised participants with COVID-19.

## Study Endpoint(s)

## Primary Endpoint

The primary endpoint is the levels of acute phase reactant CRP over 7 days follow-up.

## Secondary Endpoints

Secondary endpoints include, but are not limited to:

- - - - Levels of acute phase reactant CRP over 35 days follow-up
      - Length of hospitalisation from baseline (days)
      - Survival at Day35, mortality data collected from EPIC database for both HCs and randomised individuals
      - White blood cell count over 7 days follow-up
      - Neutrophil count over 7 days follow-up
      - Lymphocyte count over 7 days follow-up
      - Monocyte count over 7 days follow-up
      - Eosinophil count over 7 days follow-up
      - Basophil count over 7 days follow-up
      - Procalcitonin over 7 days follow-up
      - D-dimer count over 7 days follow-up
      - Blood pressure over 7 days follow-up
      - Pulse rate over 7 days follow-up
      - Temperature over 7 days follow-up
      - Respiratory rate over 7 days follow-up
      - Time on Oxygen over 7 days follow-up
      - Time on Oxygen over 35 days follow-up
      - Proportion of individuals with Pneumonia over 7 days follow-up
      - Ordinal score (WHO scoring tool) over 7 days follow-up (including randomized individuals only)
      - Proportion of individuals on Mechanical Ventilation (MV) over 7 days follow-up
      - Time on MV over 7 days follow-up
      - Length of ICU stay (hours) over 7 days follow-up
      - Proportion of individuals with Pneumonia over 35 days follow-up
      - Proportion of individuals on Mechanical Ventilation (MV) over 35 days follow-up
      - Time on MV over 35 days follow-up
      - Length of ICU stay (hours) over 35 days follow-up

## Exploratory Endpoints

Exploratory endpoints may be measured in the circulation (blood) and, when these are available, in bronchial secretions (spontaneous expectorant or routine bronchoscopy during MV). They may include, but are not limited to:

- - - - Circulating pro-inflammatory cytokines (e.g. IL-6, TNFα, IL-1β, IL-8)
      - Cell-free DNA (cfDNA)
      - Circulating histone
      - Citrullinated H3
      - NET Elisa assay
      - NET formation assay
      - Coagulation (e.g fibrin, tissue factor, Von Willebrand factor, thrombin, thromboxane A2)
      - Complement cascade (e.g C1q)
      - Haemolysis (e.g RBC lysis)
      - Expression profiling of white blood cells by RNA seq

## Statistical Hypotheses

The statistical hypotheses are defined in terms of a ‘zero effect’ i.e. no difference between the treatments; the null hypothesis. The subsequent analyses test these statements to determine how much evidence there is to support the null hypothesis. A small p-value can be interpreted as a small probability of observing the result we obtained if there really was no difference between treatments, hence leading to rejection of the null hypothesis.

## Primary null hypothesis

- - - - There is no difference in CRP levels between hospitalised COVID-19 positive individuals receiving BAC + dornase alfa and hospitalised COVID-19 positive individuals receiving BAC only.

## Secondary null hypotheses

- - - - There is no difference in secondary endpoints between hospitalised COVID-19 positive individuals receiving BAC + dornase alfa and hospitalised COVID-19 positive individuals receiving BAC only.

## Exploratory null hypotheses

- - - - There is no difference in exploratory endpoints between hospitalised COVID-19 positive individuals receiving BAC + dornase alfa and hospitalised COVID-19 positive individuals receiving BAC only.

# STUDY DESIGN

A single-site, randomised, controlled, parallel design, open-label investigation of an approved nebulised recombinant human DNase enzyme (dornase alfa) to reduce hyperinflammation in hospitalised participants with COVID-19 (the COVASE Trial: Figure 1).

Figure 1: COVASE Trial Schematic


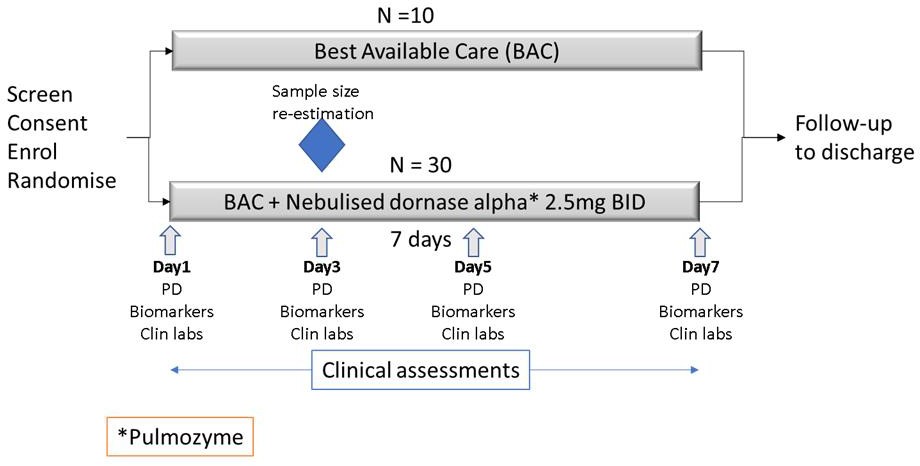


Participants will be screened, consented, enrolled and randomised up to 3 days after they are admitted to the hospital. They will be randomised in a 3:1 ratio to receive BAC + dornase alfa or BAC alone. A total of 40 participants will be enrolled (30 to receive BAC plus dornase alfa and 10 to receive BAC). On Day1 to Day7 of the trial, participants randomised to the active arm, will receive

2.5mg BID nebulised dornase alfa in addition to BAC. On Day1, Day3, Day5 and Day7, blood samples will be drawn in both trial arms in order to test pharmacodynamic endpoints (PD), biomarkers and clin labs. Clinical assessments will be undertaken daily (as per UCLH clinical guidelines). Participants will be followed until discharge or death or a maximum of 28 days follow-up.

A sample size re-estimation is planned when 12 randomised participants have completed Day7 of follow-up. This analysis will ensure that the assumptions made in the sample size calculation remain valid. However, if the variability is higher than expected then up to an additional 10 participants will be enrolled and treated with dornase alfa (up to 48 participants in total).

CRP has been chosen as the Primary Endpoint because it is a clinically important marker of inflammation and is used to make clinical treatment decisions. In addition, it is induced by the over- exuberant inflammation mediated by the NETs and inflammatory histones. CRP is a prognostic marker and correlates with clinical symptoms, inflammation and response to therapy.

Based on clinical judgement, it may be decided to keep some participants on treatment for up to 14 days. In particular, if participants have significant benefits from therapy, but show relapses of the COVID-19 inflammatory state (rising CRP and increasing oxygen requirements in the absence of bacterial infection), on completing 7 days of treatment, then the medical team have the choice of

reinstating dornase alfa treatment for up to 7 further days (14 days in total). Blood sampling, as specified, will continue until the last day of dosing with dornase alfa.

**Study controls**

Due to the evolving situation with hospitalised COVID-19 participants, the burden on the NHS and the availability of other COVID-19 trials, it is considered inappropriate to conduct a placebo- controlled study. Therefore, a randomised, controlled, open-label approach where dornase alfa is administered on top of BAC and compared to BAC alone has been adopted.

The data derived from the 10 participants who are randomised to the BAC arm of the study who do not receive dornase alfa will provide control data for all of the study endpoints.

Figure 2 Comparator Data.


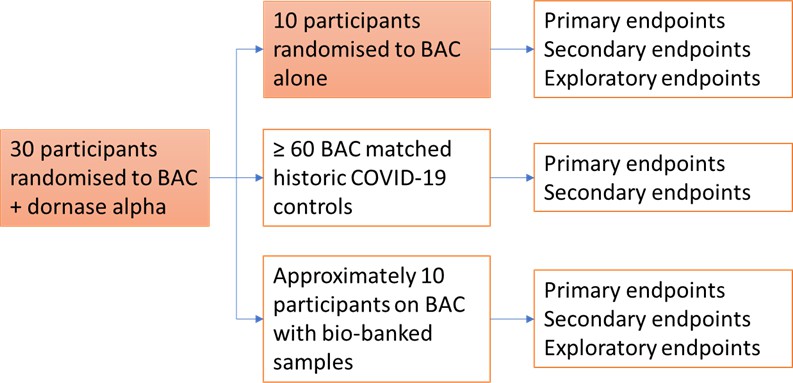

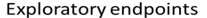


The 30 participants randomized to BAC + dornase alfa will be compared to 10 randomised controls from the COVASE study. In addition, 10 participants in other clinical trials on BAC (bio-banked samples) and 60 participants from a historic database of the first 120 people with COVID-19 treated at UCLH will also be used as comparator data.

However, to enhance the control group, a hybrid approach will be applied such that additional data will be combined with the randomised controls, illustrated in Figure 2. The sample size calculation indicates that 90 evaluable participants are required (60 control:30 active). These control data may include: data from the randomised BAC arm, historic COVID-19 UCLH database, biobanked samples from other ongoing trials and observational trials in COVID-19.

This hybrid strategy allows two things:

- provides comparators for all of the study objectives (including limited data for the exploratory objectives)
- demonstrates that the historic controls are representative/similar to the study population

Comparator data from UCLH is available as historic controls for the Primary and Secondary Endpoints. CRP is routinely measured daily (or on alternate days) in all participants admitted to UCLH and will be used to control for the Primary Endpoint. All of the Secondary Endpoints are also

routinely measured and will be available in the database. Participants in the database will be selected to act as controls as follows:

1. Apply the inclusion and exclusion criteria of the COVASE study
2. Additional selection to identify closest matches using a propensity score based on age, gender, BMI, baseline CRP (defined as the last CRP value prior to randomisation, or the first CRP following Dexamethasone for HCs) and whether they have a key comorbidity, defined as one or more of hypertension, diabetes or cardiovascular disease (see Section 11 for details).

At least 60 control participants are expected to be available to act as controls.

Other ongoing and planned trials may be a source of cytokine data as well as biobanked samples that could be used to provide control information on the exploratory endpoints (e.g. ISARIC and RECOVERY Trials) assuming suitable consent is available.

# PLANNED ANALYSES

There are two planned analyses, an interim analysis after 12 randomised individuals have completed Day7 follow-up and the final analysis, once all randomised individuals have completed follow-up (which is the minimum of discharge, death or 35 days after randomisation). At the interim analysis, only the primary endpoint and safety data will be examined, and no secondary or exploratory endpoints. A promising zones approach [1] (see Section 12 for details) will be followed at the interim analysis stage to identify the conditional power and make a decision as to whether to continue enrolment up to the planned 40 individuals randomised, or whether to increase the planned sample size up to a maximum of 48 randomised individuals. See Section 12 for details.

After the final randomised participant has completed follow-up, a more complete analysis will be undertaken, including examination of endpoints relating to the primary, secondary and exploratory objectives. A full safety analysis will also be carried out at this stage.

# SAMPLE SIZE CONSIDERATIONS

Sample size calculations were produced using the proc power function in SAS Version 9.4. These were conducted to achieve 80% power to detect a difference in the active arm versus the control group at the 5% level of significance. Based on a mean of 99mg/L in the control group and a common standard deviation of 62mg/L derived from the literature [2, 3], a total sample size of 90 participants would provide sufficient power to detect a greater than a 40% relative difference for CRP in the dornase alfa group compared to the control group. Given the reported average values in severe and non-severe participants and on clinical observations from COVID-19 patients, this difference would be achievable and clinically relevant.

This study will use existing data collected at UCLH from participants admitted with COVID-19 as a comparator group. Participants in the database will be selected to act as controls as follows:

- Apply the inclusion and exclusion criteria of the COVASE study where the appropriate data are available
- Additional selection to identify closest matches

This will give the correct ratio of active versus comparator (ratio of 1:2). To achieve the required power, this would result in 30 participants in the active treatment group and at least 60 in the control. An additional 10 participants will be recruited as a control for the exploratory objectives

and to compare the characteristics of enrolled participants with the historical controls. This gives a total of 40 participants enrolled in the study and 60 historical controls.

Participants who drop out of the study will be replaced so the sample size relates to the number of evaluable participants required.

A re-estimation of the sample size will be carried following an interim analysis when 12 randomised participants have completed Day7 follow up. This sample size calculation will be carried out using a promising zones approach [1] (see Section 12 for details).

# ANALYSIS POPULATIONS

The primary analysis will be conducted using the primary analysis population and is based on the ITT principle. Details on subjects enrolled but not included in the analysis populations will be presented as part of the CONSORT diagram.

## Primary analysis population (Intention to treat)

The primary analysis population will be all evaluable patients randomised to BAC + dornase alfa or BAC only who have at least one post-baseline CRP measurement, as well as matched historical comparators.

## Per protocol population

The per protocol (PP) population will be all evaluable patients randomised to BAC + dornase alfa or BAC only, as well as matched historical comparators, excluding important protocol violations.

Important protocol violations will be defined as:

- Initiated dornase-alfa prior to Dexmathasone.
- Withdrew from the study prior to 7 days follow-up.
- Discontinued the study drug prior to discharge from hospital or 7 days follow-up.

## Comparator population

The comparator population will be the matched historical controls and the patients randomised to BAC only.

## Safety population

The safety population will be all patients randomised to either BAC + dornase alfa or BAC only.

## Exploratory analysis population

The exploratory analysis population will be all evaluable patients randomised to BAC + dornase alfa or to BAC only, plus historical patient data from biobanked samples.

# TREATMENT COMPARISONS

The primary treatment comparison will be between individuals randomised to BAC + dornase alfa and the comparator population.

However, comparisons will also be made between the groups forming the comparator population, namely the patients randomised to BAC only and the matched historical controls. These analyses will quantify how similar the matched historical control population is to the participants randomised to BAC only, see section 8.3.1 for details.

# GENERAL CONSIDERATIONS FOR DATA ANALYSES

## Dependent Variables (Endpoint Variables)

For the primary endpoint analysis, the dependent variable will be CRP levels.

## Independent Variables

For the primary endpoint analysis, the independent variables included in the model will be treatment (dornase alfa vs. no dornase alfa), age, gender, BMI, baseline CRP level (defined as the last CRP value prior to randomisation, or the first CRP following Dexamethasone for HCs), time from baseline and whether they have a key comorbidity, defined as one or more of hypertension, diabetes or cardiovascular disease. An interaction term between treatment and time from baseline will also be included in the model.

For secondary endpoint analyses, the same covariates will be included in the models as for the primary endpoint analysis.

## Examination of Subgroups

## Primary endpoint analyses

At the interim and final analyses, the primary analysis will be to combine the two sets of individuals on BAC only in the comparator population to the individuals randomised to BAC + dornase alfa.

However, three additional analyses will be carried out at the final analysis for the primary endpoint analysis.

1. A comparison of the BAC + dornase alfa arm to those randomised to BAC only (no historical controls)
2. A comparison of the BAC + dornase alfa arm to matched historical controls (no individuals randomised to BAC only)
3. A comparison of those randomised to BAC only to matched historical controls (within control comparison).

The treatment effect estimate will be compared between the primary analysis and analyses 1. and 2. above. No formal test will be carried out to test the difference in treatment effect between the three analyses, but substantial differences will be noted.

In analysis 3., no treatment effect will be included in the model, since all individuals are on BAC only. Instead, a covariate will be included to indicate whether the individual is a historical control or a randomised individual.

In addition, analyses stratified by BAC treatment will be carried out for the primary endpoint.

## Secondary endpoint analyses

A comparison of the secondary endpoint, time on MV over 7 and 35 days follow-up, between treatment groups will be conducted only in participants that received MV.

## Multiple Comparisons and Multiplicity

This study was not powered to detect any effects relating to secondary endpoints. Therefore, the secondary analysis will involve descriptive statistics and thus there is no requirement to consider multiple comparisons in formal hypothesis testing for secondary endpoints.

# DATA HANDLING CONVENTIONS

## Premature Withdrawal and Missing Data

Mixed models will be used to analyse most endpoints; these models handle missing data naturally. Therefore, all available data will be included in all models.

## Derived and Transformed Data

Continuous endpoints will be assessed for conformance to normality and homogeneity of variance assumptions and the appropriate transformation will be conducted if necessary.

# DESCRIPTIVE STATISTICS

## Disposition of Subjects

Patient disposition will be listed. Summaries of the following patients will be presented:

- Number of individuals screened
- Number of individuals randomised
- Number of individuals who completed 7 days follow up, or follow-up to discharge from hospital, whichever occurred sooner.
- Number of individuals who withdrew consent or were lost to follow up
- Number of individuals who discontinued dornase alfa for any reason
- Number of individuals with protocol violations, to define PP population
- Number of matched historical controls or biobank samples included in the analysis

## Demographic and Baseline Characteristics

Baseline characteristics will be summarised by treatment (BAC + dornase alfa vs. BAC only) and by population: BAC + dornase alfa, randomised to BAC only, matched historical controls.

The following baseline characteristics will be presented:

- Age
- Gender
- Ethnicity
- BMI
- Baseline CRP (defined as the last CRP value prior to randomisation, or the first CRP following Dexamethasone for HCs)
- Whether they have a key comorbidity, defined as one or more of hypertension, diabetes or cardiovascular disease
- White blood cell count
- Neutrophil count
- Lymphocyte count
- Monocyte count
- Eosinophil count
- Basophil count
- Procalcitonin
- D-dimer count
- Ordinal score (WHO scoring tool), using randomized individuals only.
- Last Pre-Dexamethasone CRP (mg/L)
- Days between diagnosis and hospitalization
- Days between hospitalization and baseline
- Days between Dexamethasone initiation and baseline

# ANALYSIS CORRESPONDING TO THE STUDY OBJECTIVES

To identify individuals to include in the analysis from the historical controls cohort, the matching procedure will include an initial application of the study inclusion and exclusion criteria to identify the subjects that meet the criteria within the database. Further matching will involve the use of propensity scores to select the controls that most closely match with participants in the active treatment group. The propensity score model will be a logistic regression including all individuals randomised to dornase-alfa and all HCs, with an indicator variable as the outcome to indicate whether the individual is randomised or in the historical control cohort, and covariates included in the model for age, gender, BMI, baseline CRP (defined as the last CRP value prior to randomisation, or the first CRP following Dexamethasone for HCs), and whether they have a key comorbidity, defined as one or more of hypertension, diabetes or cardiovascular disease. The propensity matching will be done using a nearest neighbour approach.

Two controls will be matched for each participant in the active group.

As a supplementary analysis, the propensity score matching will be repeated, but instead of matching on baseline CRP as defined as the last CRP value prior to randomisation, or the first CRP following Dexamethasone for HCs, the matching will be done using the last pre-Dexamethasone CRP measurement, as well as the other factors defined above.

## Primary endpoint analysis

The primary endpoint will be compared between groups using a repeated measures mixed model, adjusted for age, gender, BMI, baseline CRP value (defined as the last CRP measurement prior to randomisation, or the first CRP following Dexamethasone for HCs), time from baseline, and whether they have a key comorbidity, defined as one or more of hypertension, diabetes or cardiovascular disease. An interaction between treatment and time from baseline will also be included in the model. CRP measurements more than 7 days after randomisation (or date of first CRP measurement following Dexamethasone initiation for HCs) will be removed from the analysis, so the comparison is over 7 days.

Due to the treatment by time interaction effect being included in the model, examining the treatment effect alone would be an inappropriate comparison, therefore the least squares means and 95% confidence intervals at the mean follow-up time will be compared between arms, which will take into account both the treatment effect and the treatment by time interaction. These means will be presented on the real scale, e.g. taking the antilog of the least squares mean if the dependent variable has been log-transformed. The difference between the least squares means (on the scale the model was fitted) and the standard error of the difference will be used to generate a treatment z-score, which will be compared against the normal distribution with mean 0 and variance 1, to obtain the two-sided p-value.

In addition, a supplementary analysis will be conducted where the area under the log(CRP) curve will be calculated for each individual up to 7 days of follow-up, divided by the number of days that the individual has been followed up for, to get a standardised area under the curve. The area will be calculated by assuming a linear line between the log(CRP) measurements, regardless of the time between measurements. If measurements are available after day 7, they will be excluded, and the last available log(CRP) measurement prior to day 7 will be used to calculate the area; for example, if the last measurement within 7 days of randomisation for a given individual was on day 4, but they have a day 10 measurement available, the area up to day 4 will be calculated, and divided by 4 to get the standardised area. A linear model will then be fitted, with standardised area under the log(CRP) curve as the outcome, adjusted for age, gender, BMI, log(baseline CRP value) (defined as the last log(CRP) measurement prior to randomisation, or the first log(CRP) following Dexamethasone for HCs) and whether they have a key comorbidity, defined as one or more of hypertension, diabetes or cardiovascular disease. A two-sided p-value will be calculated by comparing the z-score from the Wald test of the treatment effect against the normal distribution with mean 0 and variance 1.

Finally, as a supplementary analysis, the primary analysis will be performed, but including the HCs from the second propensity score matching analysis, where the matching was performed using the last pre-Dexamethasone CRP as opposed to the baseline CRP as defined above. The same model will be fitted for this analysis as the primary analysis, but the model will be adjusted for their last pre- Dexamethasone CRP as opposed to baseline CRP.

Prior to analysis, the primary endpoint will be assessed for conformance to normality assumptions and the appropriate transformation will be conducted if necessary. This model-based approach is likely to be more robust to missing or spurious data. Treatment effect will be declared significant at the 5% level of significance.

## Secondary endpoint analyses

This study was not powered to detect any effects relating to secondary endpoints. Therefore, the secondary analysis will involve descriptive statistics. In general, continuous data will be summarised using the number of individuals, mean (standard deviation), median (1st and 3rd quartiles), minimum and maximum, and categorical data will be represented as frequency counts (percentages).

A further comparison of the secondary endpoints will involve the appropriate general linear models.

For the following secondary endpoints, the same model will be fitted as for the primary endpoint, except with the dependent variable as the secondary endpoint, and adjusting for the baseline level of the secondary endpoint, as opposed to baseline CRP (baseline defined as the last measurement prior to randomisation, or the first measurement following Dexamethasone for HCs):

- Levels of acute phase reactant CRP over 35 days follow-up. The difference from the primary analysis model is that observations will be included up to 35 days after baseline. The treatment comparison will be at the mean follow-up time.
- White blood cell count over 7 days follow-up
- Neutrophil count over 7 days follow-up
- Lymphocyte count over 7 days follow-up
- Monocyte count over 7 days follow-up
- Eosinophil count over 7 days follow-up
- Basophil count over 7 days follow-up
- Procalcitonin over 7 days follow-up
- D-dimer count over 7 days follow-up
- Ordinal score (WHO scoring tool) over 7 days follow-up (using randomized individuals only)

For the following secondary endpoints, a survival analysis will be conducted. The number of events by arm will be compared. The time to event data will be censored at 28 days post last dose (Day35) for the randomised participants and at 35 days after baseline, or the date of the last electronic record, whichever is earlier, for the historical control group. If median time-to-event times exist, the Kaplan- Meier method will be used to estimate the median time-to-event times and the associated 95% confidence intervals. The survival model will include treatment as a stratification variable and significance assessed using a log-rank test. A Cox proportional hazards model will be used to generate a hazard ratio and associated confidence intervals, adjusting for age, baseline CRP value (defined as the last CRP measurement prior to randomisation, or the first CRP following Dexamethasone for HCs) and treatment as a main effect. For the time to discharge from hospital analysis, data will be censored if the participant dies prior to discharge at the date of death.

- Survival at Day35
- Time to discharge from hospital from baseline (days) to Day 35

For the following secondary endpoint, a logistic regression model will be fitted, with age, gender, BMI baseline CRP value (defined as the last CRP measurement prior to randomisation, or the first CRP

following Dexamethasone for HCs), whether they have a key comorbidity, defined as one or more of hypertension, diabetes or cardiovascular disease as covariates and treatment included as covariates.

- Proportion of individuals on Mechanical Ventilation (MV) over 7 days follow-up
- Proportion of individuals on Mechanical Ventilation (MV) over 35 days follow-up
- Proportion of individuals with Pneumonia over 7 days follow-up
- Proportion of individuals with Pneumonia over 35 days follow-up

For the following secondary endpoints, a linear regression model will be fitted, with age, gender, BMI, baseline CRP (defined as the last CRP measurement prior to randomisation, or the first CRP following Dexamethasone for HCs), whether they have a key comorbidity, defined as one or more of hypertension, diabetes or cardiovascular disease as covariates and treatment included as covariates.

In addition, a supplementary analysis will be fitted for the length of ICU stay, a logistic regression model will be fitted, with a 1 for the outcome if the individual had any hours in the ICU and a 0 if the individuals had no hours in the ICU, with age, gender, BMI, baseline CRP (defined as the last CRP measurement prior to randomisation, or the first CRP following Dexamethasone for HCs), whether they have a key comorbidity, defined as one or more of hypertension, diabetes or cardiovascular disease as covariates and treatment included as covariates.

- Length of ICU stay (hours) over 7 days follow-up
- Length of ICU stay (hours) over 35 days follow-up
- Time on Oxygen over 7 days follow-up
- Time on Oxygen over 35 days follow-up
- Length of hospitalisation from baseline (days)

For the “time on Oxygen” analyses, regardless of the amount of oxygen received, if there is evidence that any oxygen was provided to the participant on that day, 24 hours will be added to the time on Oxygen.

For the following secondary endpoints, descriptive summary statistics only will be presented:

- Blood pressure over 7 days follow-up
- Pulse rate over 7 days follow-up
- Temperature over 7 days follow-up
- Respiratory rate over 7 days follow-up
- Time on MV over 7 days follow-up. This will only include individuals who were on MV at any point over 7 days follow-up.
- Time on MV over 35 days follow-up. This will only include individuals who were on MV at any point over 35 days follow-up.

Continuous endpoints will be assessed for conformance to normality assumptions and the appropriate transformation will be conducted if necessary.

The secondary outcome analysis will use the primary analysis population.

Note: if models fail to converge or have evidence of over-fitting, covariates will be removed from the models in the following order: serious condition, Sex, Age, BMI, baseline value of measurement.

## Exploratory endpoint analyses

Exploratory analyses will not be performed by Exploristics and will be performed separately.

## Safety analyses

Safety of dornase alfa will be assessed by comparisons of adverse events (AEs), serious adverse events (SAEs), treatment-emergent adverse events (TEAEs) and deaths. The safety population is defined in Section 6.4.

All AEs will be summarized by BAC + dornase alfa/BAC only. A TEAE is an AE that starts or worsens at any time after initiation of study drug on Day1 through to end of follow-up.

For SAEs occurring in ≥5% of individuals, follow-up-adjusted event rates based on events by randomised arm will be provided. The rates will be calculated as the total number of events in the randomised arm divided by the total sum of days follow-up in that randomisation arm, multiplied by 365.25, to give the expected number of events per patient year.

Listings of all TEAEs and SAEs will be provided.

# INTERIM ANALYSIS

At the interim analysis, baseline demographics, subject disposition and primary endpoint analyses will be carried out. This will occur after the first 12 randomised individuals have completed Day7 follow-up.

A sample size re-estimation will be performed based on a promising zones approach [1]. At the interim stage, the following individuals will be included:

- approximately 9 individuals randomised to BAC + dornase alfa
- approximately 3 individuals randomised to BAC only
- approximately 18 individuals (2 × the number of individuals randomised to BAC + dornase alfa) included from the matched historical control cohorts

This gives a total of approximately 30 individuals included in the analysis. However, if there are insufficient HC data available at the interim analysis to perform propensity score matching (less than 3 × the number of individuals randomised to dornase-alfa), all HCs will be included in the interim analysis.

Prior to an interim analysis sample size re-estimation, the planned sample size at the final analysis is to include:

- 30 individuals randomised to BAC + dornase alfa
- 10 individuals randomised to BAC only
- 60 individuals (2 × the number of individuals randomised to BAC + dornase alfa) included from the matched historical control cohorts

Therefore the planned sample size is 100 individuals.

However, at the interim analysis, a sample size re-estimation will be carried out, and the overall sample size could be increased to a maximum of:

- 36 individuals randomised to BAC + dornase alfa
- 12 individuals randomised to BAC only
- 72 individuals (2 × the number of individuals randomised to BAC + dornase alfa) included from the matched historical control cohorts

Giving a maximum permitted sample size of 120 individuals.

A decision rule for whether to increase the number of individuals randomised into the trial will be derived using a promising zones approach [1]. This approach calculates the power at the interim analysis, conditional on the interim standardised treatment effect size, termed the conditional power. The conditional power at the interim stage will fall into one of three possible zones, a futility zone, a promising zone or a favourable zone.

- If the conditional power is below 0.46 it will be considered to fall into the futility zone. If the conditional power is in the futility zone at the interim analysis, it is unlikely that the final analysis will produce a p-value of <0.05 for the treatment effect, even if the sample size were increased to the maximum permitted sample size of 48 individuals randomised. Therefore, if the conditional power falls in the futility zone at the interim, recruitment will continue as planned to 40 randomised individuals and a final analysis will be performed as originally planned.
- If the conditional power is between 0.46 and 0.8, it will be considered to fall into the promising zone. In this zone, there is less than the 80% power that the study was powered for originally, but if the sample size were to be increased to somewhere between 40 and 48 randomised individuals, the power could be increased to close to 80%, and there would be an increased probability of observing a p-value of <0.05 for the treatment effect at the final analysis.
- If the conditional power is above 0.8, the conditional power will fall into the favourable zone. In this zone, there is more than the 80% power that the study was powered for originally. Therefore, recruitment will be continued to the planed 40 randomised individuals without a change to the planned sample size. There will be no decrease in planned sample size since the treatment effect at the interim analysis could be an over-estimate by chance.

# REFERENCES

1. Mehta C.R. and Pocock S.J. Adaptive Increase in Sample Size when Interim Results are Promising: A Practical Guide with Examples. Statistics in Medicine, 2011, 30(28):3267-84.
2. Han H. et. al. Profiling serum cytokines in COVID-19 patients reveals IL-6 and IL-10 are disease severity predictors. Emerging Microbes and Infections, 2020, 9(1):1123-1130.
3. Zhou F. et. al. Clinical course and risk factors for mortality of adults in participants with COVID-19 in Wuhan, China: a retrospective cohort study. Lancet, 2020, 395:1054-1062

# ATTACHMENTS

## Table of Contents for Data Display Specifications Final analysis

See TFLs_v1.6_clean.docx for final set of Tables, Figures and Listings.
